# Sex differences in cardiologic medication provision for adults with coronary heart disease: an analysis of health claims data from 2018 to 2020 in Saxony-Anhalt, Germany

**DOI:** 10.1186/s12913-024-10727-4

**Published:** 2024-03-06

**Authors:** Steffen Fleischer, Stephanie Heinrich, Gabriele Meyer, Rafael Mikolajczyk, Sara Lena Lückmann

**Affiliations:** 1 Martin-Luther-University Halle Wittenberg, Medical Faculty, Institute of Nursing and Health Science, Magdeburger Str. 8, 06108 Halle (Saale) , Germany; 2 AOK Sachsen-Anhalt, Magdeburg, Germany; 3 Martin-Luther-University Halle Wittenberg, Medical Faculty, Institute of Medical Epidemiology Biometrics and Informatics, Halle (Saale) , Germany

**Keywords:** Sex differences, Quality of healthcare, Coronary heart disease, Cardiologic healthcare, Health claims data, Use of statins

## Abstract

**Background:**

Coronary heart diseases (CHDs) have experienced the largest increase worldwide as a cause of death, accounting for 16% of all deaths. In Saxony-Anhalt, a federal state in Germany, both CHD morbidity and acute myocardial infarction mortality rates are particularly high. Several risk factors associated with CHDs have been studied in Saxony-Anhalt, but sex differences in service use and medication have not been investigated. This study therefore aimed to investigate sex differences in the quality and quantity of cardiological care provided to adults with CHD.

**Methods:**

This study used health claims data from 2018 to 2020 to analyse the utilisation of healthcare services and adherence to medication-related guideline recommendations in primary and specialist care. The sample included 133,661 individuals with CHD from a major statutory health insurance company (Germany).

**Results:**

Almost all CHD patients (> 99%) received continuous primary care. Continuous cardiologist utilisation was lower for females than for males, with 15.0% and 22.2%, respectively, and sporadic utilisation showed greater differences, with 33.5% of females and 43.4% of males seeking sporadic cardiologist consultations. Additionally, 43.1% of the identified CHD patients participated in disease management programmes (DMPs). The study also examined the impact of DMP participation and cardiologist care on medication uptake and revealed that sex differences in medication uptake, except for statin use, were mitigated by these factors. Statins were prescribed to 42.9% of the CHD patients eligible for statin prescription in accordance with the QiSA indicator for statin prescription eligibility. However, there were significant sex differences in statin utilisation. Female CHD patients were less likely to use statins (35.2%) than male CHD patients were (50.1%). The difference in statin utilisation persisted after adjustment for DMP participation and cardiologist consultation.

**Conclusions:**

This study highlights sex differences in the utilisation of cardiological healthcare services for patients with CHD in the Saxony-Anhalt cohort. These findings underscore the continuing need for interventions to reduce sex inequalities in accessing healthcare and providing health care for patients with CHD. Factors at the health care system, patient, and physician levels should be further investigated to eventually improve statin prescription in people with CHD, especially women.

## Introduction

Cardiovascular diseases (CVDs) are the leading cause of death worldwide and in Germany [1, 2]. CVDs include coronary heart disease (CHD), heart failure, and acute myocardial infarction (AMI). Despite a decline in new cases of CHD in Germany, the aging population has led to an increase in CHD cases [1]. CHD has experienced the largest increase worldwide as a cause of death, accounting for 16% of all deaths [2].

In Saxony-Anhalt, a federal state in Germany, both CHD morbidity and AMI mortality rates are particularly high [3]. High blood pressure, a major risk factor for CHD, affects one-third of the adult population in Germany [4]. Its prevalence increases with age and is more than 31% in people older than 45 years and more than 60% in people older than 65 years [4]. Saxony-Anhalt not only has one of the highest CHD morbidity rates but also has the highest AMI mortality rate in Germany [1]. With an average age of 47.9 years in 2022, Saxony-Anhalt had the oldest population of all German federal states [5].

Socioeconomic factors, lifestyle choices, and disparities in healthcare provision contribute to increased cardiac morbidity [6, 7]. For several years, Saxony-Anhalt has consistently been the German state with the highest mortality rate from ischaemic heart disease. An analysis of disease-related and socioeconomic risk factors in Saxony-Anhalt, published in 2014, showed the first or second highest burden among German states for all relevant risk factors considered [8]. A subsequent study came to similar conclusions, showing differences in the distribution of risk factors between male and female patients in Saxony-Anhalt [9]. Studies have shown that higher numbers of cardiologists and internal medicine inpatient beds and fewer residents per chest pain unit are associated with a lower lifetime incidence of CVD [6]. However, no association could be found with participation in disease management programmes (DMPs) in a Saxony-Anhalt sample [9, 10]. DMPs are evidence-based treatment programmes for people with chronic diseases that aim to improve the coordination of care, include regular follow-up checks, as well as counselling and training for patients [11]. They were introduced in Germany in 2002.

Saxony-Anhalt has an absolute 9% lower utilisation rate of cardiological care by specialist physicians than does the average of all other federal states [3], although a medium density of cardiologists can be assumed in Saxony-Anhalt compared to the federal territory [1]. However, given the higher mean age of Saxony-Anhalt population, this may not be sufficient.

Physicians often underestimate symptoms of disease, such as the frequency of angina pectoris attacks [12], and in Germany, almost half of patients diagnosed with CHD are not treated according to guideline recommendations [13]. In particular, women have a lower treatment rate associated with cardiovascular medications [14] and are less likely to receive evidence-based treatment for ischaemic heart disease than men are, resulting in worse health outcomes [15, 16]. In addition, therapy adherence is often limited, with the use of medical therapy decreasing in the year following an AMI [17].

Responsible use of medicines (RUM), as understood by the WHO, means that patients receive the right medicines at the right time, use them appropriately and benefit their health outcomes [18]. A variety of quality indicators for assessing RUM exist and are used internationally to evaluate service delivery across medical disciplines and different diseases [19]. AOK, the largest statutory health insurance company in Germany, utilises disease-specific performance and medication-related quality indicators for outpatient care to improve and compare quality in primary and ambulatory care; these indicators are known as QiSA indicators [20]. Eight disease-specific sets of indicators are currently in use and have been developed and validated using consensus methods. These indicators are intended for implementation at the practice level and are calculated as the proportion of individuals with a specific condition who receive particular interventions or diagnostics and recommended thresholds. There is no obligation to use a set of quality indicators or to publish results of such a quality assessment in Germany.

Adherence to guideline recommendations that do not directly affect the prescription of drugs is considered a separate quality indicator and, if meaningful, should have at least some impact on RUM by increasing the indicated use of medicines and reducing the inappropriate use of drugs. In the case of CHD in Germany, guideline recommendations exist for continuity of primary care, specialist involvement (i.e., cardiologists), and participation in a DMP [21].

Regional variability and sex-specific differences in cardiac care are known and have been described in various contexts. Differences have been observed in the utilisation of DMPs, in specialist care provided by cardiologists, and in drug treatment, which mostly disadvantage female patients and patients living in structurally weak regions [3, 14, 22–25]. Within Saxony-Anhalt, there are also regional differences in the age- and sex-standardised prevalence of CHD at the county level, ranging from 15.0 to 21.8% [3].

The question of whether participation in DMP or cardiology care can improve the quality of RUM and reduce existing sex differences in the treatment of CHD patients remains unanswered. Therefore, we examined RUM using QiSA in adults aged 18 years and older with CHD in a cohort of AOK insureds in Saxony-Anhalt based on claims data. In particular, we investigate gender differences and how they can be mitigated by DMP participation and specialist involvement.

The study aimed to answer the following questions:

1. What are the sex differences in primary and cardiologist care for people with CHD in Saxony-Anhalt?
2. Is there an association between adherence to available guidelines (e.g., enrolment in a DMP, cardiologist involvement) and medication uptake in CHD patients?
3. How do DMP and cardiologist care influence sex differences in medication uptake?

## Methods

The study involved an analysis of anonymised claims data from AOK Saxony-Anhalt (AOK SAN), a major statutory health insurance company. Approximately 2.2 million people live in Saxony-Anhalt [26], 796,000 of whom had health insurance with AOK SAN in 2020 [27], representing 36.2% of the population in Saxony-Anhalt. In terms of age and sex distribution, the sample can be considered representative of Saxony-Anhalt but not of Germany, as Saxony-Anhalt has a completely different demographic structure than the rest of Germany.

The sample included all persons who were continuously insured by the AOK SAN from 2018 to 2020, who were at least 18 years old by 31 December 2020, and who were diagnosed with CHD in 2018. Patients were not excluded if they were continuously insured with the AOK SAN before the date of death. The place of residence had to be in Saxony-Anhalt by 31 December 2020 or at the time of death.

The obtained health claims data included anonymised sociodemographic data, primary care data, hospital data, and data on prescribed drugs. Furthermore, several variables were defined and generated using the provided data, as presented in Table 1 (i.e., diagnoses, QiSA indicators for CHD, and care utilisation). Accounted ICD-10 diagnoses were used to categorise insured persons. QiSA indicators are intended to be quality indicators for assessing the quality of drug prescription for selected diseases. The percentage of patients who met the predefined eligibility criteria and received a certain drug was calculated as follows: $p = \frac{{{\rm{drug\ a\ prescribed}}}}{{{\rm{eligible\ for\ prescription\ of\ drug\ a}}}} \times 100$. The use of services was operationalised by recorded visits to physicians, namely, general practitioners and cardiologists. Depending on annual visits, service use was classified as continuous, sporadic or no service utilisation.

**Table 1:** Variable definitions

Variable	Operational definition
CHD (yes/no)	ICD-10-GM I20-I25
AMI (yes/no)	ICD-10-GM I21-I23, I25.2
Care dependent (yes/no)	Formal care grade present, as evaluated
DMP participation/utilisation (yes/no)	Registration in any DMP
Continuous primary care (yes/no)	At least one consultation either with a general practitioner or a cardiologist per year in the years 2018, 2019, and 2020
Cardiologist treatment: continuous utilisation	Cardiologist consultation every year in 2018, 2019, and 2020
Cardiologist treatment: sporadic utilisation	At least one cardiologist consultation in 2018, 2019 or 2020, but not in every year
Cardiologist treatment: no utilisation	No cardiologist consultation in 2018, 2019 or 2020
QiSA indicator 6 beta-blocker prescription with heart failure (eligible yes/no)	Stable CHD (ICD-10-GM I25) and heart failure (ICD-10-GM I50), excluding pulmonary heart disease (ICD-10-GM I27) or asthma (ICD-10-GM J45)
QiSA indicator 6 beta-blocker prescription with heart failure (prescribed yes/no)	Relevant drugs: ATC-codes C07
QiSA indicator 7 beta-blocker prescription after acute myocardial infarction (eligible yes/no)	Stable CHD (ICD-10-GM I25) and acute myocardial infarction (ICD-10-GM I21-I23, I25.2, hospital diagnosis), excluding myocardial infarction that occurred more than two years ago, or diagnosed with asthma (ICD-10-GM J45)
QiSA indicator 7 beta-blocker prescription after acute myocardial infarction (prescribed yes/no)	Relevant drugs: ATC-codes C07
QiSA indicator 8 statin prescription (eligible yes/no)	Eligible patients with CHD (ICD-10-GM I20-I25), excluding patients > 73 years and a diagnosed ischemicischemic cardiomyopathy (ICD-10-GM I25.5), and patients with diabetes type II (ICD-10-GM E11) and terminal renal insufficiency (ICD-10-GM N18.5)
QiSA indicator 8 statin prescription (prescribed yes/no)	Relevant drugs: ATC-codes C10AA, C10BA and/or C10BX
QiSA indicator 9 ACE inhibitors or AT-II antagonists prescription with heart failure and/or high blood pressure (eligible yes/no)	Eligible patients with stable CHD (ICD-10-GM I25), and heart failure (ICD-10-GM I50) and/or high blood pressure (ICD-10-GM I10-I15), excluding patients with pulmonary heart disease (I27)
QiSA indicator 9 ACE inhibitors or AT-II antagonists prescription with heart failure and/or high blood pressure (prescribed yes/no)	Relevant drugs: ATC-codes C09A, C09B,C09C and/or C09D

### Statistical analysis

The data were analysed descriptively using absolute and relative frequencies for count data and means and standard deviations for continuous data. Crude proportions as cumulative incidences were compared using risk difference (RD). Group comparisons of count data were performed with generalised linear models by calculating odds ratios (ORs). Generalised linear models were fitted for the QiSA criteria, with drug prescription as the dependent variable (prescribed/not prescribed), cardiologist consultation (cardiologist/no cardiologist), DMP (DMP/no DMP), and sex (male/female) as the independent variables. Only subsamples were used to fit the models that fulfilled the eligibility criteria for the relevant QiSA indicator. Where applicable, 95% confidence intervals were calculated. The data were provided and anonymised through a trusted person from the AOK SAN.

Ethical approval was granted by the Ethics Committee of the Faculty of Medicine, Martin Luther University Halle-Wittenberg (approval number: 2021 − 215). Given that the analysis included claims data, individual informed consent for participation in the study was not needed. This study was also approved by the aforementioned ethics committee. Data protection was ensured in accordance with European and German data protection legislation. All the data were anonymised before analysis, and the results are presented in aggregate statistics only.

## Results

### Sample description

The study sample included all 133,661 people with CHD in 2018. The sample characteristics are shown in Table 2. The sample represented 22.8% of all individuals with statutory health insurance from the AOK SAN who had continuous insurance since 2018, were at least 18 years old in 2020 (*n* = 584,029) and were diagnosed with CHD in 2018. In the study sample, one in four persons required long-term care assistance. Almost all had a confirmed diagnosis of high blood pressure, more than one-third had heart failure, and almost 2% had experienced an acute myocardial infarction within the previous year.

**Table 2:** Characteristics of the study sample in 2018

	Individuals with CHD in 2018 (*n* = 133,661)
Age, (mean, sd)	76.9 ± 12.3
Female, n (%)	70,352 (52.6)
High blood pressure^a^, n (%)	125,692 (94.0)
Heart failure^b^, n (%)	47,350 (35.4)
Acute myocardial infarction in 2018^c^, n (%)	2,439 (1.8)
Care dependent^d^, n (%)	34,437 (25.8)

^a^ ICD-10-GM I10-I15, ^b^ ICD-10-GM I50, ^c^ ICD-10-GM I21-I23, I25.2, hospital data only, ^d^ Having a formal German care grade (1 or higher)

### Outpatient health care utilisation and medication indicators

Continuous primary care was defined as at least one consultation with either a general practitioner or a cardiologist per year in 2018, 2019, and 2020 was displayed by almost all CHD patients (*n* = 133,292/133,661; 99.7%).

The utilisation rates of cardiologist treatment were lower for continuous utilisation with 18.4% (*n* = 24,632/133,661) and sporadic utilisation with 38.2% (*n* = 51,096/133,661). A sex difference was apparent for continuous utilisation, with 15.0% (*n* = 10,553/70,352) female CHD patients and 22.2% (*n* = 14,079/63,309) male CHD patients receiving continuous cardiologist consultation (RD = 7.2; 95% CI: 6.8; 7.7). This difference was more pronounced for sporadic cardiologist consultations, with 33.5% (*n* = 23,577/70,352) of female CHD patients compared to 43.4% (*n* = 27,492/63,309) of male CHD patients (RD = 9.9; 95% CI: 9.4; 10.4) having sporadic cardiologist consultations.

Of all identified CHD patients, 43.1% (*n* = 57,557/133,661) took part in a DMP in the years 2018, 2019 or 2020. Additionally, a smaller proportion of women with CHD (39.0%, *n* = 27,424/70,352) took part in DMP than male CHD patients (47.6%, *n* = 30,133/63,309), with a RD of 8.6 (95% CI: 8.1; 9.1).

The raw risk differences in medication indicator performance are displayed in Table 3.

**Table 3:** QiSa medication indicator performance raw risk differences by sex

	Female	Male	Total	RD; CI (95%))
Indicator 6 Patients with heart failure and prescribed beta blocker	75.2% (*n* = 13,890/18,462)	77.5% (*n* = 13,920/17,964)	76.3% (*n* = 27,810/36,426)	RD = 2.3; CI: 1.4; 3.1
Indicator 7 Patients after acute myocardial infarction and prescribed beta blocker	85.1% (*n* = 702/825)	86.3% (*n* = 1,063/1,232)	85.8% (*n* = 1,765/2,057)	RD = 1.2; CI: -1.9; 4.3
Indicator 8 eligible Patients with prescribed statins	35.2% (*n* = 15,383/43,666)	50.1% (*n* = 19,321/38,531)	42.2% (*n* = 34,704/82,197)	RD = 14.9; CI: 14.2;15.6
Indicator 9 eligible Patients with heart failure and/or high blood pressure and prescribed ACE-inhibitors or AT-II antagonists	77.2% (*n* = 44,653/57,823)	80.6% (*n* = 42,668/52,921)	78.8% (*n* = 87,321/110,744)	RD = 3.4; CI: 2.9; 3.9

RD = risk difference; CI = confidence interval

### Associations between medication indicators and health care utilisation and sex

Participation in a DMP and cardiologist consultation were associated with an increased appropriate prescription rate of the indicated drugs in the sample, as shown in Table 4. The differences in prescription rates between nonparticipants and participants for DMP for beta blockers in CHD patients with heart failure, beta blockers in CHD patients after myocardial infarction, and ACE inhibitors or AT-II antagonists in CHD patients with heart failure or high blood pressure were similar for both female and male patients. This approach was also used for the comparison of patients without a cardiologic consultation and patients with a cardiologic consultation. There were additional differences in statin prescription between female and male patients.

**Table 4:** Medication indicators met by DMP participation, cardiologist consultation and sex

		DMP participation		Cardiologist consultation	
	Sex	No N (%)	Yes N (%)	NoN (%)	YesN (%)
Indicator 6 Patients with heart failure and prescribed beta blocker	F	6,817 (72.1)	7,073 (78.6)	8,987 (71.4)	4,903 (83.6)
	M	5,547 (73.7)	8,373 (80.3)	7,004 (71.4)	6,916 (84.8)
Indicator 7 Patients after acute myocardial infarct and prescribed beta blocker	F	348 (85.3)	354 (85.5)	509 (83.0)	193 (92.3)
	M	454 (84.7)	609 (88.6)	704 (84.4)	359 (92.3)
Indicator 8 eligible Patients with prescribed statins	F	7,575 (27.4)	7,808 (48.7)	10,070 (30.3)	5,313 (50.8)
	M	7,475 (37.1)	11,846 (64.5)	10,894 (41.5)	8,427 (68.7)
Indicator 9 eligible Patients with heart failure and/or high blood pressure and prescribed ACE-inhibitors or AT-II antagonists	F	25,797 (75.6)	18,856 (79.6)	33,009 (75.3)	11,644 (83.2)
	M	20,928 (79.1)	21,740 (82.2)	26,957 (77.7)	15,711 (86.3)

The numbers represent the absolute numbers of persons with prescriptions and the percentages of persons eligible for prescription

For all four investigated medication indicators, a generalised linear model was fitted, with the prescription of the drug as the dependent variable (prescribed/not prescribed) and with cardiologist consultation (cardiologist/no cardiologist), DMP (DMP/no DMP), and sex (male/female) as the independent variables. Table 5 shows the coefficients and confidence intervals. According to the additive models, for indicators 6, 7 and 9, cardiologist consultation had the greatest effect, followed by participation in a DMP. Sex had the least impact in these models.

**Table 5:** Prescription of the indicated medication depending on service utilisation and sex (generalised linear models)

		Odds Ratio	CI (95%)
Indicator 6 Patients with heart failure and prescribed beta blocker	Cardiologist (ref. no cardiologist)	1.10	1.09; 1.11
	DMP (ref. no DMP)	1.06	1.05; 1.07
	Male (ref. female)	1.00	0.99; 1.01
Indicator 7 Patients after acute myocardial infarction and prescribed beta blocker	Cardiologist (ref. no cardiologist)	1.09	1.06; 1.12
	DMP (ref. no DMP)	1.02	0.99; 1.05
	Male (ref. Female)	1.01	0.98; 1.04
Indicator 8 eligible Patients with prescribed statins	Cardiologist (ref. no cardiologist)	1.14	1.14; 1.15
	DMP (ref. no DMP)	1.25	1.24; 1.26
	Male (ref. Female)	1.12	1.11; 1.13
Indicator 9 eligible Patients with heart failure and/or high blood pressure and prescribed ACE-inhibitors or AT-II antagonists	Cardiologist (ref. no cardiologist)	1.06	1.06; 1.07
	DMP (ref. no DMP)	1.03	1.03; 1.04
	Male (ref. female)	1.02	1.02;1.03

CI = Confidence interval

The presence of statins (indicator 8) had the greatest effect on all three independent variables, with participation in a DMP and cardiologic consultation showing the greatest effect on prescription. Sex had a substantial effect on prescription, indicating that women are even less likely to receive statin prescriptions, irrespective of whether they participated in a DMP or had a cardiologic consultation.

## Discussion

Our analysis showed that there are differences between men and women with CHD in the use of DMP and cardiologist care. The difference was more pronounced for consultations with cardiologists than for consultations with a DMP. This is in opposition to total specialist consultations in Germany, where women exceed men by 15% across specialist groups [28]. Additionally, for the four investigated QiSA indices, a difference in the frequency of the indicated prescriptions between male and female patients was observed, with female patients being at a disadvantage. However, the magnitude of these differences varies considerably and is particularly apparent for the use of statins.

For QiSA indicators 6, 7, and 9, the effect attributable solely to patient sex was negligible compared to that attributed to care received through DMPs or from cardiology specialists. The confidence intervals for indicators 6 and 7 do not point in a clear direction, and the effect is rather small for indicator 9. However, even after controlling for the type of care received (DMP and/or cardiology consultation), the sex of patients had a stable impact on the frequency of indicated statin prescriptions (indicator 8).

The low frequency of indicated statin prescriptions is particularly problematic and lower than that reported in other studies, especially for a high-income country [29–31]. The utilisation of DMP or cardiologist consultations clearly promoted the prescription of statins. However, women still receive less adequate treatment. Specialist care and DMP seem to do little to reduce this sex difference in statin prescription, although they substantially increase the rate of prescription. Sex differences in statin prescription have been described for cardiovascular diseases and are well established [29, 32, 33]. The use of statins for the primary prevention of CVD has been subject to debate [34–36], and statins may even have continuing effects on prescription rates for secondary prevention. Women consistently exhibit lower lipid control rates than men [30]. The reasons for this sex difference still remain unclear, but women reported, to a lesser degree, that their physician has offered them to take statins and more often declined or discontinued statin prescription [29]. The role of physicians in primary care seems especially important considering the increase in CHD patients without statin prescription 6 months after hospital discharge in Germany and the overall low proportion of CHD patients receiving high-intensity statin therapy compared to those in other Western countries [37]. The efficacy and effectiveness of statins for the secondary prevention of cardiovascular diseases are well established for myocardial infarction, death from CVD, and overall death in both sexes [38]. Furthermore, evidence suggests that even older patients with polypharmacy do not benefit from the discontinuation of statins [39]. The reasons for the lower uptake of statin medication in female patients with CHD are attributed to the lower probability of receiving a cardiologist consultation and more adverse reactions to statin therapy in women [40].

Low utilisation of statin medications is likely a multifaceted issue with several contributing factors. A significant portion of CHD patients may not fully understand the role of statins in secondary prevention. This lack of awareness about the benefits of statin therapy may contribute to low utilisation rates. Variations in statin prescription rates among healthcare providers in Germany might arise from differences in clinical judgement and adherence to treatment guidelines. Some physicians may be more cautious in prescribing statins due to concerns about potential side effects, while others might be more proactive in recommending them. The structure of the German healthcare system can also influence statin utilisation rates. Factors such as reimbursement policies and access to specialist care services might impact patient adherence to statin therapy. The adverse effects of statins, including muscle pain and liver abnormalities, are a concern for both patients and healthcare providers. However, it is essential to distinguish between legitimate concerns and exaggerated fears. Although statins have potential side effects, these side effects are generally rare and often outweighed by their cardiovascular benefits. In some cases, misinformation or media reports may contribute to an exaggerated perception of these side effects. Healthcare providers should engage in open and transparent discussions with patients to address their concerns and provide reassurance when necessary. In some cases, patients may be reluctant to start statin therapy due to concerns about side effects, as discussed earlier. It is crucial for healthcare providers to engage in shared decision-making with patients, present them with all available treatment options and discuss the potential benefits and risks. This approach empowers patients to make informed choices about their cardiovascular care. Additionally, tools and resources for healthcare providers to facilitate these discussions should be developed and encouraged. Shared decision-making not only addresses patient concerns but also fosters a sense of partnership in the treatment process, which can enhance medication adherence.

### Strengths and limitations

Our study has several strengths. We included a large sample that represented more than a third of the Saxony-Anhalt population and were able to use data for a three-year observation period. This enabled us to base our analyses on a broad database and to make reliable statements on medication and health service use for the target population. Furthermore, we investigated factors associated with CHD morbidity and mortality in Saxony-Anhalt that were not investigated in previous publications.

Nonetheless, our study has limitations due to the origin and scope of the data. It uses claims data from one statutory health insurance in one federal state in Germany only. The data serve providers for billing of services rendered, so data that do not serve the purpose of billing are not transmitted to the health insurance. In addition, only services that have been used are counted; these services do not always correspond to prescribed services. Thus, it cannot be determined whether the lower utilisation of cardiologist treatment or certain drugs is due to a lack of referral/prescription or nonredemption of the referral/prescription. However, it seems unlikely that the low rates and differences in the prescription of statins are based on this potential source of error, as the other three indicators show completely different magnitudes. Therefore, it is not sufficiently plausible based on this mechanism why the frequency of statin prescriptions deviates to this extent from a 70.9% prescription rate in high-income countries [31]. We did not analyse antiplatelet drug prescription in our study, although it is included as a QiSA criterion since claims data for acetylsalicylic acid prescriptions in Germany are not available for the vast majority of patients, as most have to pay for them out of pocket.

## Conclusion

In general, the results suggest that the care of patients with CHD in structured programmes such as DMP or by cardiology specialists is associated with improved medication management. Against this backdrop, the lower utilisation of these specialised services by female patients appears especially critical and to be questioned. Therefore, research on possible inhibiting factors at the level of healthcare provision and sex-specific differences in the use of DMP and cardiological healthcare services is highly important. From a system perspective in Germany, a higher rate of DMP participation and cardiologist consultation should be pursued to increase prescription rates in people with CHD.

In summary, low statin utilisation rates in Germany are influenced by a complex interplay of factors involving patient information, physician practices, healthcare system structure, and patient concerns about adverse effects. Addressing these issues requires a multifaceted approach that includes improved patient information, physician training, healthcare system reforms, and honest discussions about the true risks and benefits of statin therapy. This approach can help enhance cardiovascular care and reduce the burden of heart disease and stroke in Saxony-Anhalt.

Investigating these variations is critical to understanding why statin utilisation rates are low in certain regions or types of healthcare facilities. Additionally, interventions such as physician education programmes can promote evidence-based prescribing practices and improve the consistency of statin prescription rates across the country.

## Data Availability

The datasets generated and/or analysed during the current study are not publicly available due to data protection laws regarding individual health claims data in Germany.

## Abbreviations

AMI: Acute myocardial infarction
AOK SAN: AOK Saxony-Anhalt
CHD: Coronary heart disease
CI: Confidence interval
CVD: Cardiovascular disease
DMP: Disease management programme
OR: Odds ratio
RD: Risk difference
RUM: Responsible use of medicine
